# [Retracted] Evaluation of the role of human β-defensin 3 in modulation of immunity and inflammatory response after knee replacement

**DOI:** 10.3892/etm.2025.12900

**Published:** 2025-06-02

**Authors:** Jiguang Jia, Hao Peng, Sen Chen

Exp Ther Med 13:1343–1346, 2017; DOI: 10.3892/etm.2017.4100

Following the publication of the above paper, it was drawn to the Editor’s attention by a concerned reader that, regarding the flow cytometric plots shown in Fig. 1 on p. 1344, the data panels shown for Fig. 1B and D appeared to show similar groupings of dots, which would not have been anticipated if these experiments had been performed discretely under different experimental conditions, suggesting a fundamental flaw either in the way in which these experiments were performed, or in how the results were outputted.

The Editor of *Experimental and Therapeutic Medicine* has decided that this paper should be retracted from the Journal on account of a lack of confidence in the presented data. The authors were asked for an explanation to account for these concerns, but the Editorial Office did not receive a reply. The Editor apologizes to the readership for any inconvenience caused.

